# Early metoprolol use in ICU patients with congestive heart failure is associated with increased 30-day mortality: a causal machine learning study

**DOI:** 10.3389/fphar.2026.1771969

**Published:** 2026-02-09

**Authors:** Yunzhu Chen, Tianyou Li

**Affiliations:** 1 Zunyi Fourth People’s Hospital, Zunyi, Guizhou, China; 2 Bojishan Hospital, Jinan, Shandong, China

**Keywords:** causal forest, causal machine learning, congestive heart failure, metoprolol, treatment heterogeneity

## Abstract

**Background:**

The risk-benefit balance of early metoprolol administration in critically ill patients with congestive heart failure (CHF) admitted to the intensive care unit (ICU) remains controversial. However, high-quality causality clinical evidence is still lacking. This study utilized real-world data and causal forest algorithm to investigate the average treatment effect (ATE) of early metoprolol use on 30-day mortality in ICU patients with CHF. We also explored the heterogeneity of individual treatment effects (ITE) to identify clinical characteristics across different treatment-effect populations.

**Method:**

Study population and their clinical characteristics were obtained from the MIMIC-IV database (version 3.1). Propensity score matching (PSM) was performed to balance baseline confounders. Causal forest models estimated the ATE and ITE. Variable importance, conditional average treatment effect (CATE), and ITE-based quartile analysis were performed to identify heterogeneity and clinical characteristics of patients.

**Results:**

A total of 5,758 patients were included in the initial cohort, with 3,934 patients entering the final analysis after PSM. The causal forest model indicated that early metoprolol use increased the risk of 30-day ICU mortality by 2% compared with no use group (ATE = 0.02, 95% CI: 0.0004–0.0396, P = 0.045). APSIII, age, and SOFA were the top three variables contributing to the ATE. The causal forest model estimated the conditional average treatment effects for every variable. Individual treatment effects exhibited a right-skewed distribution. The highest ITE percentile was associated with APSIII >64, age >78 years, SOFA >8, WBC >13.95 × 10^9^/L, and BUN >43.06 mg/dL.

**Conclusion:**

Early metoprolol use within 24 h of ICU admission in CHF patients is associated with increased 30-day mortality. Therapeutic effects were heterogeneous, with the adverse effects being particularly pronounced in patients with high APSIII, advanced age, elevated SOFA, high white blood cell counts, and elevated blood urea nitrogen levels. These findings suggest careful consideration should be given to the early use of metoprolol in high-risk patients.

## 1 Introduction

Congestive heart failure (CHF), characterized by impaired cardiac pumping function or association with elevated ventricular filling pressures, is one of the most prevalent cardiac diseases among the elderly (Benjamin et al., 2019; AHA/ACC/HFSAACC/AHA Joint Committee Members, 2022). CHF affects over 26 million individuals globally, with increasing prevalence observed annually. Prevention and management of these diseases have consequently emerged as a major focus within clinical medicine and public health (Singh et al., 2024; Chioncel et al., 2019).

β-blockers (such as metoprolol, bisoprolol, and carvedilol) exert biological functions that reduce ejection fraction and improve ventricular remodeling, serving as cornerstone therapies for heart failure (Paolillo et al., 2021; Beghini et al., 2025). Numerous evidence-based medical studies indicate that long-term administration of beta-blockers suppressed an overactive sympathetic nervous system, improve ventricular remodeling, and significantly reduce mortality and readmission rates in patients with chronic heart failure (Martínez-Milla et al., 2019). The 2022 AHA guidelines for heart failure management indicate that metoprolol exerts a protective effect in heart failure with reduced ejection fraction and in cases of prolonged tachycardia combined with congestive heart failure. It is recommended as a first-line treatment (Heidenreich et al., 2022). However, the risk-benefit balance of metoprolol in critically ill patients admitted to the ICU with CHF remains controversial. The pathophysiological perspective suggests that CHF patients in the ICU often remain hemodynamically unstable and reliant on sympathetic tone for compensatory output. At this time, the negative inotropic and negative chronotropic effects of metoprolol could theoretically disrupt this compensatory mechanism, potentially leading to hypoperfusion and worsened organ function, outweighing long-term benefits (Schurtz et al., 2023).

This clinical dilemma persists due to a lack of high-quality evidence. Randomized controlled trials (RCTs) in critically ill patients confront ethical and practical limitations. Existing evidence mostly derives from observational studies, while the conclusions of the few relevant RCTs are inconsistent, making it difficult to establish reliable guidance (Zhou et al., 2025; Ibanez et al., 2025; Munkhaugen et al., 2025). Thus, decisions regarding whether to initiate and when to commence beta-blocker therapy in the ICU setting remain unresolved clinical problems (Jondeau et al., 2009; Bhagat et al., 2019).

Causal machine learning, an emerging methodology, offers a new perspective for resolving the aforementioned challenges. It utilizes counterfactual inference methods combined with the powerful computational capabilities of machine learning to perform causal inference. This approach allows for better use of large amounts of real-world data. Unlike traditional machine learning, which focuses on predicting outcome probabilities, causal machine learning concentrates on uncovering causal effects where altering treatment conditions leads to outcome changes (Feuerriegel et al., 2024). The causal forest is a classic causal machine learning algorithm, offering robust stability and interpretability, and capable of detecting data heterogeneity (Liu M. et al., 2025). It is an ideal tool for realizing precision medicine and moving research from ‘average changes’ towards ‘individualized alterations’.

In summary, this study employed real-world data and causal forest algorithms to investigate the effect of early initiation of metoprolol administration on 30-day mortality among ICU patients with CHF and to identify subgroups benefiting or harmed by treatment. This study aims to address clinical questions and provide theoretical guidance for clinical practice.

## 2 Materials and methods

### 2.1 Study population

Data of study population were obtained from the MIMIC-IV database (version 3.1). The MIMIC database is a publicly available clinical database of intensive care unit (ICU) patients, jointly developed by the Massachusetts Institute of Technology and Beth Israel Deaconess Medical Center in Boston, USA, and that it contains single-center ICU patient data from this hospital (Johnson et al., 2023). The diagnostic information in the database was derived from de-identified diagnostic records in the electronic medical system of Beth Israel Deaconess Medical Center, and that all diagnoses were made by ICU physicians based on clinical conditions of patients. Author Yunzhu Chen completed the course required for database access and has been granted permission to download research data (certification number: 73859939). The MIMIC database has implemented patient privacy protection measures. Consequently, our research has been granted ethical exemption. This research was performed in compliance with the STROBE.

**FIGURE 1:**
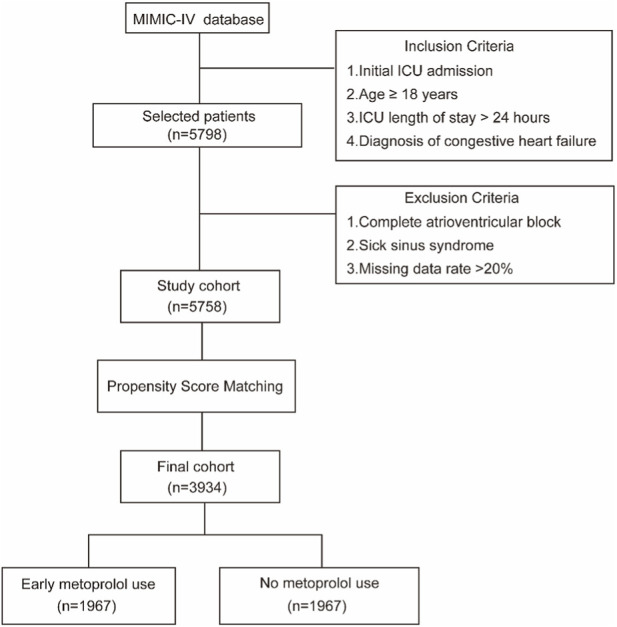
Flowchart for patient enrolment in the study.

The inclusion criteria for this study were: (1) Congestive heart failure as the primary diagnosis for admission to the ICU (ICD-9: 428.0); (2) First-time ICU admission; (3) Age over 18 years; (4) ICU stay exceeding 24 h. Exclusion criteria: (1) Complete atrioventricular block (ICD-10: I44.2); (2) Sick sinus syndrome (ICD-10: I49.5); (3) Missing data rate of study variables exceeding 20%. Early metoprolol use was defined as metoprolol therapy administered within 24 h of ICU admission; No metoprolol use referred to the nonuse of metoprolol and other beta-blocker agents within 24 h of ICU admission. A total of 5,758 patients were enrolled in the initial study cohort by fitting the aforementioned criteria. After PSM, 3,934 patients were ultimately included in the final study cohort (Figure 1), comprising 1,967 patients in the early metoprolol use group and 1,967 in the no metoprolol use group.

### 2.2 Data extraction

Data of enrolled CHF patients were extracted from the MIMIC-IV database (version 3.1) using PostgreSQL (version 14.0). Research variables comprised seven sections: (1) Demographic information including age, gender, weight; (2) Comorbidities including hypertension, diabetes mellitus, myocardial infarction, liver disease, kidney disease, chronic respiratory disease, cerebrovascular disease; (3) Invasive operations including CPR, invasive vent; (4) Critical illness assessment scores: including APS III, SOFA; (5) Vital signs: including heart rate, systolic blood pressure, diastolic blood pressure, mean arterial pressure, oxygen saturation (SO_2_), arterial oxygen partial pressure (PO_2_), arterial carbon dioxide partial pressure (PCO_2_); (6) Pharmacological interventions: including norepinephrine, spironolactone, angiotensin converting enzyme inhibitors (ACEI), angiotensin receptor blockers (ARB); (7) Laboratory test results: including white blood cell count (WBC), blood urea nitrogen (BUN), partial thromboplastin time (PTT), blood glucose, troponin I, hemoglobin. The aforementioned indicators were first measured within 24 h of the patient’s admission to the ICU. To eliminate potential bias, subjects with variable missingness exceeding 20% would be excluded.

### 2.3 Clinical outcome

The clinical outcome in this study was all-cause mortality within 30 days of ICU admission.

### 2.4 Statistical analysis

The normality of continuous variables was assessed using the Shapiro-Wilk test. Variables that followed a normal distribution were presented as mean ± standard deviation and were compared between groups using the t-test. For non-normally distributed variables, data were expressed as median (interquartile range) and were compared using the Mann–Whitney U test. Categorical data were expressed as percentages and analyzed using chi-square tests. Missing data were imputed through the random forest algorithm. The PSM employed 1:1 optimal matching using all baseline covariates listed in Table 1. The Causal Forest algorithm was performed to estimate the ATE, CATE, variable importance to ATE, ITE and the interaction between important variables. The hyperparameters of the study model were (1) num.trees = 1,000; (2) mtry = 4; (3) sample.fraction = 0.7; (4) honesty = TRUE. To further investigate treatment heterogeneity, all patients were divided into four groups based on their ITE values. To further investigate treatment heterogeneity, all patients were divided into four groups based on their ITE values. Logistic regression analysis was employed to examine mortality risk factors in subgroups. Comparisons of key covariates between subgroups were conducted using t-tests or non-parametric tests. Statistical analysis was performed using the R software (version 4.5.1). P-value <0.05 was considered statistically significant.

**TABLE 1:** Baseline characteristic of study population before and after PSM.

Characteristic	Before matching			After matching		
	Nonusers^a^ (n = 3,791)	Metoprolol users^a^ (N = 1,967)	P value^b^	Nonusers^a^ (n = 1967)	Metoprolol users^a^ (n = 1,967)	P value^b^
Gender						
Female	1,721 (45%)	892 (45%)	>0.9	905 (46%)	892 (45%)	0.7
Male	2,070 (55%)	1,075 (55%)		1,062 (54%)	1,075 (55%)	
Age	76 (65, 84)	77 (66, 85)	0.016	76 (66, 85)	77 (66, 85)	0.4
Weight	80 (66, 95)	78 (65, 95)	0.12	79 (66, 95)	78 (65, 95)	0.6
Hypertension						
No	2,276 (60%)	1,023 (52%)	<0.001	1,043 (53%)	1,023 (52%)	0.5
Yes	1,515 (40%)	944 (48%)		924 (47%)	944 (48%)	
Diabetes						
No	2,252 (59%)	1,160 (59%)	0.8	1,141 (58%)	1,160 (59%)	0.5
Yes	1,539 (41%)	807 (41%)		826 (42%)	807 (41%)	
Myocardial infarct						
No	2,776 (73%)	1,234 (63%)	<0.001	1,333 (68%)	1,234 (63%)	<0.001
Yes	1,015 (27%)	733 (37%)		634 (32%)	733 (37%)	
Liver disease						
No	3,369 (89%)	1,856 (94%)	<0.001	1,820 (93%)	1,856 (94%)	0.02
Yes	422 (11%)	111 (5.6%)		147 (7.5%)	111 (5.6%)	
Renal disease						
No	2,322 (61%)	1,304 (66%)	<0.001	1,283 (65%)	1,304 (66%)	0.5
Yes	1,469 (39%)	663 (34%)		684 (35%)	663 (34%)	
Chronic pulmonary disease						
No	2,210 (58%)	1,132 (58%)	0.6	1,130 (57%)	1,132 (58%)	>0.9
Yes	1,581 (42%)	835 (42%)		837 (43%)	835 (42%)	
Cerebrovascular disease						
No	3,325 (88%)	1,684 (86%)	0.025	1,683 (86%)	1,684 (86%)	>0.9
Yes	466 (12%)	283 (14%)		284 (14%)	283 (14%)	
APSIII	47 (36, 60)	44 (34, 56)	<0.001	45 (35, 56)	44 (34, 56)	0.3
SOFA	6 (4, 9)	5 (3, 7)	<0.001	5 (3, 8)	5 (3, 7)	0.003
Invasive vent						
No	1,880 (50%)	1,074 (55%)	<0.001	1,025 (52%)	1,074 (55%)	0.12
Yes	1,911 (50%)	893 (45%)		942 (48%)	893 (45%)	
CPR						
No	3,671 (97%)	1,932 (98%)	0.002	1,917 (97%)	1,932 (98%)	0.1
Yes	120 (3.2%)	35 (1.8%)		50 (2.5%)	35 (1.8%)	
Norepinephrine						
No	2,810 (74%)	417 (21%)	<0.001	990 (50%)	417 (21%)	<0.001
Yes	981 (26%)	1,550 (79%)		977 (50%)	1,550 (79%)	
Spironolactone						
No	3,668 (97%)	1,892 (96%)	0.3	1,903 (97%)	1,892 (96%)	0.3
Yes	123 (3.2%)	75 (3.8%)		64 (3.3%)	75 (3.8%)	
ARB						
No	3,724 (98%)	1,914 (97%)	0.02	1,917 (97%)	1,914 (97%)	0.8
Yes	67 (1.8%)	53 (2.7%)		50 (2.5%)	53 (2.7%)	
ACEI						
No	3,512 (93%)	1,733 (88%)	<0.001	1,775 (90%)	1,733 (88%)	0.031
Yes	279 (7.4%)	234 (12%)		192 (9.8%)	234 (12%)	
Heart rate (bpm)	84 (73, 97)	88 (76, 103)	<0.001	87 (76, 100)	88 (76, 103)	0.12
Systolic blood pressure (mmHg)	119 (103, 137)	123 (108, 140)	<0.001	124 (107, 143)	123 (108, 140)	0.4
Diastolic blood pressure (mmHg)	62 (51, 74)	65 (55, 77)	<0.001	64 (53, 78)	65 (55, 77)	0.12
Mean arterial pressure (mmHg)	77 (67, 89)	81 (71, 93)	<0.001	81 (70, 93)	81 (71, 93)	0.5
PO2 (mmHg)	124 (78, 314)	122 (77, 321)	0.9	121 (76, 315)	122 (77, 321)	0.4
PCO_2_ (mmHg)	41 (35, 48)	41 (36, 48)	0.15	41 (36, 48)	41 (36, 48)	0.6
SO2 (%)	97 (94, 98)	97 (94, 98)	0.5	97 (94, 98)	97 (94, 98)	0.2
WBC (×10^9^/L)	10.4 (7.3, 14.6)	10.3 (7.7, 14.1)	>0.9	10.3 (7.4, 14.2)	10.3 (7.7, 14.1)	0.6
BUN (mg/dL)	28 (18, 46)	25 (17, 39)	<0.001	25 (17, 39)	25 (17, 39)	0.3
PTT(s)	33 (29, 41)	33 (28, 43)	0.7	33 (28, 41)	33 (28, 43)	0.6
Troponin I (ng/mL)	0.08 (0.03, 0.34)	0.11 (0.04, 0.52)	<0.001	0.09 (0.03, 0.38)	0.11 (0.04, 0.52)	<0.001
Blood glucose (mg/dL)	123 (100, 155)	123 (102, 156)	0.2	125 (102, 159)	123 (102, 156)	0.4
Hemoglobin (g/dL)	10.80 (9.40, 12.30)	11.20 (9.80, 12.70)	<0.001	11.10 (9.70, 12.50)	11.20 (9.80, 12.70)	0.032

^a^ n (%); Median (Q1, Q3).

^b^ Pearson’s Chi-squared tests; Wilcoxon rank sum test.

## 3 Result

### 3.1 Baseline of the study population

**FIGURE 2:**
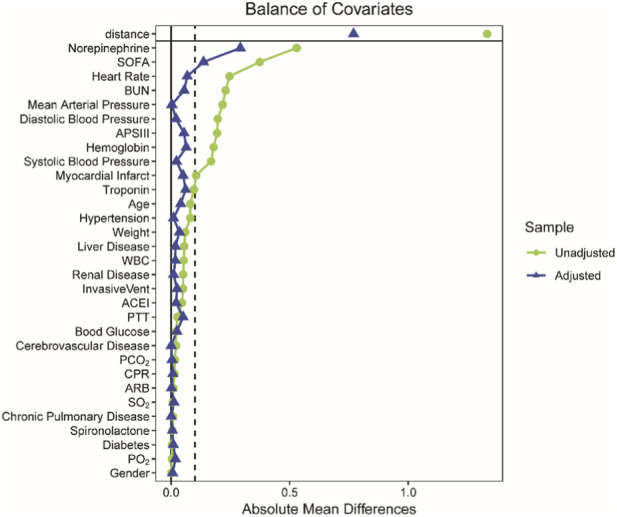
The line chart of absolute standardized mean difference of all variables before and after PSM.

Before PSM, the study data exhibited significant imbalance. The P-values of 20 variables were less than 0.05 in intergroup comparisons with ten variables of Standardized Mean Difference (SMD) greater than 0.1. After 1:1 optimal PSM matching, the imbalances between groups were effectively improved. There were 7 variables with intergroup comparison p-values below 0.05, with only norepinephrine and SOFA exhibiting SMDs greater than 0.1. There were seven variables with P-value less than 0.05 in intergroup comparisons. Only norepinephrine and SOFA had the SMD greater than 0.1. Moreover, the medians of the aforementioned variables were approximately similar between groups after matching. The causal forest model could effectively mitigate the impact of variable imbalance on outcomes (f), and both variables held high clinical significance. Consequently, all variables were involved into subsequent analyses after PSM (Table 1; Figure 2).

### 3.2 ATE of the intervention

The causal forest model estimated the ATE of early metoprolol use on 30-day mortality. Result of ATE was 0.02 (95% CI = 0.0004–0.0396; P = 0.045), which suggested early metoprolol use resulted in an approximately 2% increase in the risk of 30-day mortality of whole final population. The number needed to harm for this intervention was 50 (NNH = 50).

### 3.3 Heterogeneity analysis of ATE

**FIGURE 3:**
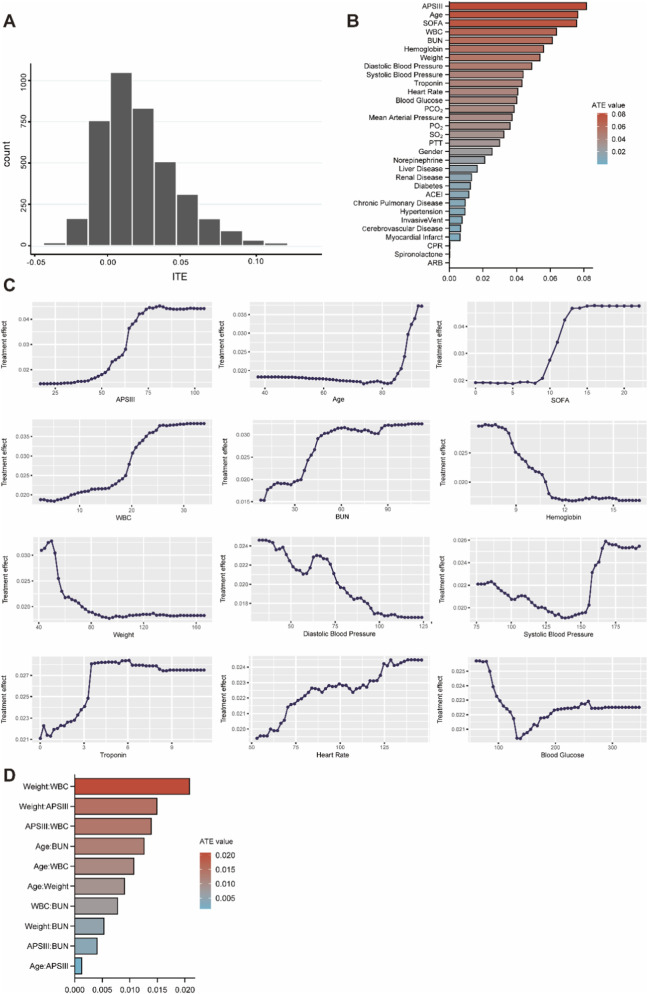
The results of ATE Heterogeneity analysis. **(A)** ITE distribution histogram. **(B)** Bar chart showing the ranking of contribution of variables to ATE. **(C)** Partial dependency plot of CATE for key variables. **(D)** Bar chart of variable interaction ranking.

ITE histogram revealed a right-skewed asymmetric distribution of study population, which suggested treatment heterogeneity (Figure 3A). Variable importance analysis identified APSIII, age, SOFA, WBC, and BUN as the top five variables contributing to ATE (Figure 3B). We selected variables with ATE contribution values higher than 0.03 for conditional average treatment effect (CATE) analysis. The results were visually represented through partial dependence plots, which illustrated how ATE changed with variable values (Figure 3C). Body weight and WBC were the highest interaction effect variable pairs, with 2.1% contribution to ATE attributable to their interaction. The interaction effects of all other variable pairs were less than 2% (Figure 3D).

### 3.4 Heterogeneity analysis of ITE

**FIGURE 4:**
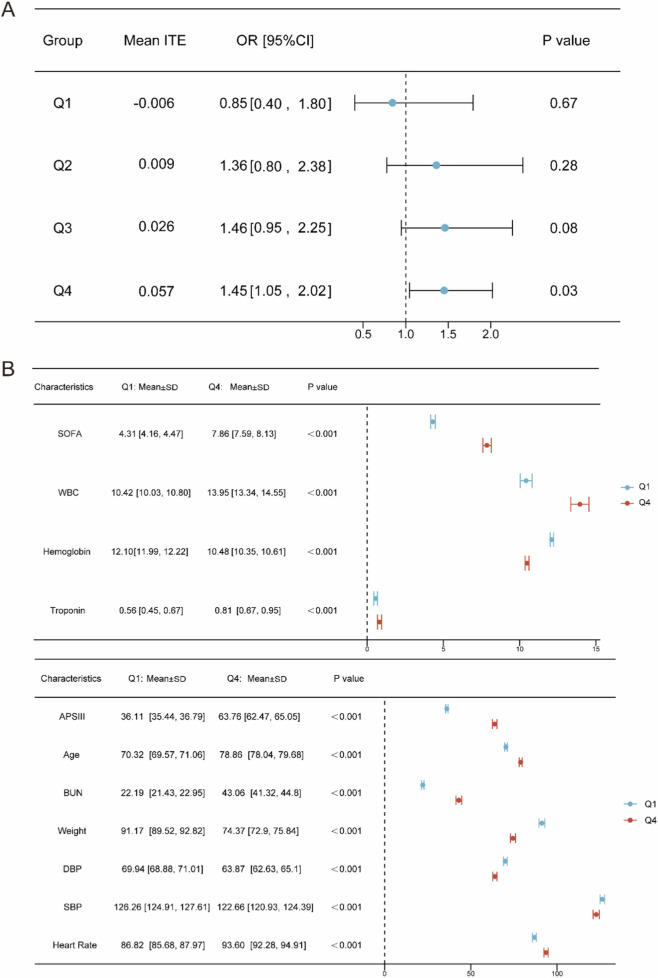
The results of ATE heterogeneity analysis. **(A)** Forest plot of logistic regression results of group Q1–Q4. **(B)** Forest plot of results of key variable comparisons between Q1 and Q4 groups.

To further elucidate the heterogeneity of ITE, we quartile-stratified the early metoprolol use group and no metoprolol use group according to their ITE values, then merged the respective quartiles to form the final groups (Q1-Q4). In Q1 (mean ITE = −0.006), metoprolol use had an non-significant protective effect (OR = 0.85, 95% CI: 0.40–1.80, P = 0.67). In Q4 (mean ITE = 0.057), metoprolol use significantly increased mortality risk (OR = 1.45, 95% CI: 1.05–2.02, P = 0.03) (Figure 4A).

We found that key variables of top 10 contribution to ATE differed significantly between Q4 and Q1 (Figure 4B). For the purpose of directly describing the characteristics of high-risk individuals, we extracted the mean values of Q4 group variables as cut-off points and employed CATE as the reference of variable range. Results indicated that patients with CHF on their first ICU admission who presented with an APS III > 64, age > 78 years, SOFA > 8, WBC > 13.95 × 10^9^/L, and BUN > 43.06 mg/dL were at significantly increased risk of 30-day mortality when given metoprolol early.

### 3.5 Model sensitivity analysis

Calibration test revealed that the estimates of mean.forest.prediction and differential.forest.prediction yielded close to 1, with P-values below 0.05 (Table 2). This indicated the model was correctly established and possessed a robust capacity for identifying heterogeneity. Furthermore, we adjusted the hyperparameters of the causal forest model by setting num.trees = 2000. The ATE of refitting model was 0.02 (95% CI = 0.0004–0.0396), similar with the model presented model in our study, which suggests the model was robust.

**TABLE 2:** Results of the calibration test of causal forest model.

Examination parameters	Estimate	Std. Error	t value	P value
mean.forest.prediction	0.97	0.53	1.83	0.03
differential.forest.prediction	1.13	0.52	2.16	0.01

To investigate the therapeutic window for metoprolol administration, we extended the dosing window to within 48 h and fitted a causal forest model using the same methodology. Results showed ATE = 0.005 (95% CI: −0.015–0.027; P = 0.62), with eight of the top ten variables contributing to ATE remaining identical (Supplementary Table S1). These findings indicate that the period within 24 h of ICU admission exhibits greater specificity for intervention risk regarding 30-day mortality, though high-risk patient characteristics remain broadly similar regardless of timing.

We further applied the same causal forest model to analyze the impact of early metoprolol use on in-hospital mortality, aiming to more comprehensively evaluate the short-term effects of early intervention. Results showed an ATE of 0.012 (95% CI: −0.006–0.003; P = 0.18), with the top 10 variables by ATE ranking consistent with the primary analysis (Supplementary Table S2). These sensitivity analyses indicated that the heterogeneity of treatment effects exhibits high robustness and consistency.

## 4 Discussion

This study utilized real-world data and causal machine learning algorithms to investigate whether early metoprolol use in ICU patients diagnosed with congestive heart failure influenced 30-day mortality. We found that in the overall study population, early metoprolol use was associated with a 2% increase in 30-day mortality (ATE = 0.02, P = 0.045, NNH = 50). This effect exhibited significant heterogeneity, with individual treatment effects showing a right-skewed distribution, which implied that for a substantial proportion of patients, the harm effect far exceeds the average 2% mortality. Our study successfully identified the variables contributing most to the ATE and high-risk patient subgroups. The marginal statistical effect was observed for the ATE (ATE = 0.02, P = 0.045). Specifically, we believe this effect could be due to several factors, including the potential influence of single-center data on statistical power, as well as the dilution of the average treatment effect by treatment heterogeneity. The top five key variables and their characteristics were: higher ICU scores (APS III > 64, SOFA > 8), older age (> 78 years), and increased markers of inflammation (WBC >13.95 × 10^9^/L) and renal dysfunction (BUN > 43.06 mg/dL).

The study population comprised a heterogeneous cohort of patients hospitalized for acute decompensated heart failure, with baseline blood pressure resembling the intergroup mean, reflecting this diversity. However, deeper analysis revealed that patients’ hemodynamic status served as a key modifier of the early metoprolol treatment effect. Although routine vital signs may not have shown extreme abnormalities, the need for norepinephrine support and higher SOFA cardiovascular scores signaled underlying hemodynamic instability and poorer physiological reserve (Vincent et al., 1996). Our causal inference analysis indicates that it is precisely within this patient subgroup that early metoprolol therapy shows the most significant association with increased mortality risk. This suggests that metoprolol’s negative inotropic and negative chronotropic effects may expose or exacerbate their hemodynamic vulnerability. Therefore, the focus of clinical decision-making should shift to “whether the current hemodynamics and overall physiological state of this heart failure patient can tolerate such therapy.” The comprehensive high-risk features identified in this study—such as elevated SOFA scores and APS III—provide quantitative support for this assessment (Di Santo et al., 2021).

In the present stage, several clinical guidelines recommend the long-term use of beta-blockers for chronic heart failure (Ostrominski et al., 2024). Our findings indicate that early administration of metoprolol increases mortality among ICU inpatients diagnosed with congestive heart failure. This appears to contradict prevailing views, but we believe the numerous characteristics of this study may serve as a valuable supplement to the mainstream perspective.

Firstly, the study population has been expanded: classic RCT studies are constrained by ethical limitations, making it difficult to conduct research on critically ill patients in ICUs that may cause significant harm (Paddock et al., 2022). Real-world studies can fill this gap in such populations. Beyond investigating ATE, our research focuses more on the heterogeneity of characteristics in populations at risk from early metoprolol use. Patients with higher APS-III and SOFA presented with more severe conditions and poorer prognoses (Fan and Ma, 2024; Liu C. et al., 2025). Those with APS-III > 64 and SOFA > 8 might confront critical conditions such as hypotension, shock, and multiple organ injury (Ranzani et al., 2025; Özdemir et al., 2021). Initiating treatment with beta-blockers, exemplified by metoprolol, at this stage might compromise cardiac compensatory mechanisms, thereby contributing to adverse outcomes (Pollesello et al., 2025). Research indicated that elderly heart failure patients often experience worsening conditions and poor outcomes due to factors such as overlapping comorbidities, diminished organ reserve, and psychosocial vulnerability (Sen et al., 2025). The indiscriminate use of metoprolol might exacerbate these pathophysiological processes. Elevated white blood cell counts indicate systemic inflammatory responses potentially associated with worsening heart failure (Meier et al., 2020). Severe heart failure might reduce circulating blood volume, impair renal function, and increase blood urea nitrogen (BUN) levels (Tang et al., 2024). These mechanisms suggested partially explain high mortality outcomes in high-risk patient receiving metoprolol.

Secondly, statistical tools have become significantly more powerful. This study is focused on causal inference, we believe that the level of clinical evidence generated is higher than that of typical machine learning retrospective studies. For this reason, we specifically selected these validated key variables to assess their contribution to the ATE and to examine the distribution of these variables following the classification of study cases based on ITE. With advances in statistical theory and increased computational power, machine learning algorithms were applied more extensively across various types of medical research. They now possessed the capability to utilize counterfactual reasoning within given real-world data, enabling relatively accurate estimation of outcome changes resulting from treatment modifications (Richens et al., 2020). Causal machine learning algorithms could leverage their inherent capability to process complex, high-dimensional data, enabling more efficient and stable handling of diverse real-world datasets. They provided powerful tools for uncovering genuine causality (Weberpals et al., 2025). Currently, most real-world data studies examining the relationship between beta-blockers and heart failure employ retrospective analyses and outcome prediction machine learning methods (Wang et al., 2024; Wu et al., 2025). Our research provided valuable supplementary evidence at the causal level for this clinical problem.

Thirdly, the translation of research findings has become more convenient. Nowadays, the gold standard for clinical causal research is RCT, whose outcomes reflect differences in overall intervention effects (Chan et al., 2025). Interestingly, numerous cases have emerged within RCT studies where results from other studies of the same design prove inconsistent. The accumulation of heterogeneity within study populations has probably one key factor contributing to these divergent outcomes (Verstraete et al., 2023). Our research explored the heterogeneity of intervention effects concealed beneath the ATE framework. The heterogeneity of the experimental population was confirmed through a combined approach by grouped ITE values and logistic analysis. Building upon this foundation, we have also identified specific clinical characteristics of high-risk populations, reflecting the principles of precision medicine (Hartl et al., 2021). Concurrently, our study provides intuitive guidance for clinical practice.

This study employed causal machine learning models to not only estimate ATE but, more importantly, to reveal the underlying heterogeneity of effects. The robustness of this finding was strongly supported by sensitivity analyses (VanderWeele and Ding, 2017). Although the ATE of early metoprolol use on extended 48-h medication use and in-hospital mortality was consistent with the primary analysis, it did not reach statistical significance. The revealed pattern of individual treatment effect heterogeneity—i.e., the core clinical characteristics determining risk levels—highly aligned with findings from the 30-day mortality analysis. This consistency across outcomes at different time windows strongly suggested that patient subgroups defined by indicators such as APS III, age, SOFA, WBC, and BUN may universally exhibit greater vulnerability to early beta-blocker therapy within the ICU. Thus, the value of our study extended beyond assessing statistical significance for a single endpoint. It lied in providing a stable, transferable set of risk stratification characteristics that offer an evidence-based foundation for more refined treatment decisions within the highly heterogeneous ICU environment (Wu et al., 2020).

Although this study has obtained some findings, it still has certain limitations. Our data were derived from real-world database, the cases possibly exhibited biases, and some key variables (diagnosis and classification of heart failure, echocardiogram results, some types of therapeutic drugs) were incompletely recorded, meaning confounding factors could not be entirely ruled out. The findings remain of borderline statistical significance. Although the MIMIC database contains a large number of cases, it remains a single-center dataset, and our conclusions require validation in external cohorts. Furthermore, this study did not address the optimal timing for initiating beta-blocker therapy. These problems warrant further investigation in future research.

## 5 Conclusion

In summary, our study found that early metoprolol administration within 24 h of ICU admission for CHF diagnosed patients were associated with increased 30-day mortality, and this effect exhibited considerable heterogeneity. The adverse effect was particularly in patients with high illness severity scores, advanced age, higher WBC, and elevated BUN. These findings suggested that metoprolol should be used cautiously in critically ill heart failure patients following thorough assessment of their condition and mitigation of risk factors.

## Data Availability

The original contributions presented in the study are included in the article/Supplementary Material, further inquiries can be directed to the corresponding author.
